# Id Proteins Regulate Capillary Repair and Perivascular Cell Proliferation following Ischemia-Reperfusion Injury

**DOI:** 10.1371/journal.pone.0088417

**Published:** 2014-02-07

**Authors:** David Lee, Shantheri Shenoy, Yezina Nigatu, Matt Plotkin

**Affiliations:** Department of Medicine, Renal Research Division, New York Medical College, Valhalla, New York, United States of America; UCL Institute of Child Health, United Kingdom

## Abstract

Acute kidney injury (AKI) results in microvascular damage that if not normally repaired, may lead to fibrosis. The Id1 and 3 proteins have a critical role in promoting angiogenesis during development, tumor growth and wound repair by functioning as dominant negative regulators of bHLH transcription factors. The goal of this study was to determine if Id proteins regulate microvascular repair and remodeling and if increased Id1 expression results in decreased capillary loss following AKI. The effect of changes in Id expression *in vivo* was examined using Id1−/−, Id3RFP/+ (Id1/Id3 KO) and Tek (Tie2)-rtTA, TRE-lacz/TRE Id1 (TRE Id1) mice with doxycycline inducible endothelial Id1 and β-galactosidase expression. Id1 and 3 were co-localized in endothelial cells in normal adult kidneys and protein levels were increased at day 3 following ischemia-reperfusion injury (IRI) and contralateral nephrectomy. Id1/Id3 KO mice had decreased baseline capillary density and pericyte coverage and increased tubular damage following IRI but decreased interstitial cell proliferation and fibrosis compared with WT littermates. No compensatory increase in kidney size occurred in KO mice resulting in increased creatinine compared with WT and TRE Id1 mice. TRE Id1 mice had no capillary rarefaction within 1 week following IRI in comparison with WT littermates. TRE Id1 mice had increased proliferation of PDGFRβ positive interstitial cells and medullary collagen deposition and developed capillary rarefaction and albuminuria at later time points. These differences were associated with increased Angiopoietin 1 (Ang1) and decreased Ang2 expression in TRE Id1 mice. Examination of gene expression in microvascular cells isolated from WT, Id1/Id3 KO and TRE Id1 mice showed increased Ang1 and αSMA in Id1 overexpressing cells and decreased pericyte markers in cells from KO mice. These results suggest that increased Id levels following AKI result in microvascular remodeling associated with increased fibrosis.

## Introduction

Following acute kidney injury, kidney interstitial cells become activated in response to cytokines and growth factors secreted by injured epithelial and endothelial cells and infiltrating inflammatory cells. Fibroblast activation results in remodeling of the extracellular matrix that promotes repair of damaged tubules and peritubular capillaries. With severe or irreversible injury, this process is persistently activated, resulting in tissue fibrosis, capillary rarefaction and chronic renal failure [1]. Recent studies have demonstrated that endothelial cells and pericytes that form the peritubular microvasculature are a source of injury induced fibroblasts and myofibroblasts that produce extracellular matrix [2]. The molecular mechanisms responsible for this, however, are not well understood.

During the normal adaptive process for repairing tissue damage, TGFβ and BMP signals regulate cell proliferation and differentiation. In the adult kidney, BMPs are predominately produced by medullary tubular epithelial cells. Following ischemia-reperfusion injury, BMP7 expression initially decreases [3] but then increases in regenerating tubular cells in the outer medulla, peaking at days 1–3 [4]. BMP signal transduction is mediated by nuclear effector R-Smads, with downstream activation of regulatory factors including Id proteins [2], [3]. The four Id protein isoforms (Id1–4) are dominant negative regulators of bHLH transcription factor driven cell differentiation. bHLH proteins are key regulators of lineage and tissue specific gene expression. By inhibiting bHLH activity, Id proteins inhibit differentiation and have been shown to have a key role in maintaining stem and progenitor cell fate during development and in both normal adult tissues and tumors [5], [6].

Id levels are transiently increased by BMP in numerous cell types including endothelial cells [7], [8]. Id expression must be downregulated for terminal differentiation as demonstrated by *in vitro* studies using Id overexpression in mesenchymal progenitor cells [9]. The role of Id1 and Id3 in mesenchymal cell phenotype regulation *in vivo* has been clearly demonstrated in cardiac valve formation, where increased endothelial Id1 and 3 expression in response to myocyte BMP2 and 4 secretion is required for endothelial-mesenchymal transition (EndMT) and cell migration with formation of the cardiac jelly or matrix needed for valve formation [10]. Mice with endothelial cell specific knockout of the Bmpr1a (Alk3) receptor display deficient endothelial mesenchymal transition and absence of Id1 and 3 expression and die with cardiac valve agenesis [11].

Id1 and Id3 are highly expressed in endothelial cells in both developing and tumor blood vessels. Endothelial Id levels are undetectable in most quiescent adult tissues. The role of Id proteins in embryonic vasculogenesis and tumor angiogenesis has been extensively examined by both *in vitro* and *in vivo* genetic studies [6]. Knockout of both Id1 and Id3 results in embryonic lethality due to vascular and cardiac malformations. Loss of function studies in Id+/−/Id3−/− mice demonstrate normal development but impaired tumor angiogenesis and tumor growth [12]. Endothelial cell specific Id1−/− and id1−/−, Id3−/− mice demonstrate impaired dermal wound healing associated with decreased angiogenesis [13] and granulation tissue [26]. Studies of Id1−/− or Id3−/− mice have also shown defects in the stromal microenvironment resulting in defects in inflammatory cell homing and matrix production [14]. Few studies however, have examined the role of these proteins in adult tissue homeostasis and non-malignant diseases. The role of Id proteins in angiogenesis and regeneration in response to adult kidney injury remains unknown.

Based on the accumulating evidence for the role of Id proteins in angiogenesis and mesenchymal cell fate determination including endothelial-mesenchymal transition and endothelial paracrine signaling, we hypothesized that changes in microvascular Id expression levels following kidney injury may regulate both vessel repair and contribute to the development of interstitial fibrosis as a result of changes in mesenchymal cell phenotype. To determine the role of Id proteins in vascular remodeling and repair in the kidney, we examined expression patterns of Id 1 and 3 in normal kidney and following ischemia-reperfusion injury (IRI) and differences in microvascular repair and interstitial fibroblast responses in wild type, Id1−/−, Id3+/− and mice with inducible transgenic endothelial cell Id1 overexpression. Results in this study demonstrate that Id proteins regulate capillary growth and remodeling following kidney injury with decreased capillary rarefaction and increased medullary peritubular fibroblast proliferation and fibrosis resulting from Id1 overexpression.

## Materials and Methods

### Mouse Strains

*Id1^−/−^* mice (C57BL/6 background), were obtained from Dr. Robert Benezra, and were described previously [15]. The *Id3RFP/+* ^−^ mouse strain *B6;129S-Id3^tm1Pzg^/J* was obtained from Jackson Labs and backcrossed with C57BL/6 mice. The *Id1^−/−^Id3RFP/+* mice were generated through crossing of *Id1^+/−^* and *Id3RFP/+*. Transgenic mice with Id1 transcriptionally regulated by a tetracycline responsive element (TRE) operator were obtained from Dr. Robert Benezra, and crossed with Tg(Tek-rtTA,TRE-lacZ)1425Tpr/J mice from Jackson Labs to create a strain (*TRE-Id1*) with a Tie2 (Tek) expression dependent Id1 Tet-On phenotype. Both mouse strains are C57BL/6 congenic. Mice were genotyped by performing PCR on DNA obtained from tail biopsy, using the appropriate published primer combinations. Mice were fed doxycycline containing pellets (625 mg/kg, TD.08541, Harlan Labs, Madison, WI) beginning at 1 week prior to IRI to induce Id1 expression or standard chow and remained on these diets throughout the study period for all experiments.

All mice were housed in accordance with guidelines from American Association for Laboratory Animal care. Research protocols and procedures were evaluated and approved by the New York Medical College Institutional Animal Care and Use Committee and followed accepted guidelines for laboratory mouse welfare.

### Ischemia-reperfusion Injury

8–10 week old male mice were anesthetized with ketamine and xylazine. Mice were placed on a heating pad at 37C. The left renal hilum was identified and the renal vessels were dissected and clamped for 35 minutes. The vessels of the right kidney were ligated with sutures and the kidney was subsequently removed. Kidney ischemia and reperfusion were verified by expected changes in tissue color. The midline incision was closed with 3-0 silk suture. Mortality for all genotypes was <10%. 5–7 mice for each genotype were used for each time point.

### Quantitative PCR

RNA was extracted from kidneys using an RNeasy Mini Kit (Qiagen, Valencia, CA) and quantified by spectrophotometry. cDNA was prepared by reverse transcription of 1 µg of RNA using the AB High Capacity RNA-to-cDNA Kit (Life Technologies, Grand Island, NY). Quantitative real-time PCR was performed with PerfeCTa SYBR Green Fastmix (Quanta, Surrey, UK) and the Mx3000 Real-Time PCR system (Stratagene, La Jolla, CA). mRNA levels were calculated using the ΔΔCt method, using 18S as a reference gene. Primers were designed based on published sequences for 18S, Ang1, Ang2, alpha smooth muscle actin (αSMA), and PDGF beta receptor (PDGFRβ) and are listed in Table 1.

**Table 1:** 

Gene	Forward	Reverse
18S	AGTTCCAGCACATTTTGCGA	TCATCCTCCGTGAGTTCTCC
Ang1	AGGCTTGGTTTCTCGTCAGA	TCTGCACAGTCTCGAAATGG
Ang2	CCTCGACTACGACGACTCAGT	TCTGCACCACATTCTGTTGGA
αSMA	GGCACCACTGAACCCTAAGG	ACAATACCAGTTGTACGTCCAGA
PDGFRβ	TTCCAGGAGTGATACCAGCTT	AGGGGGCGTGATGACTAGG

### X-gal Staining

10 µm frozen sections and cultured cells were fixed with 0.2% gluteraldehyde for 10 minutes and incubated in buffer containing: PBS, 5 mM K ferricyanide, 5 mM K ferrous cyanide, 0.01% sodium deoxycholate, 0.02% NP-40, 1 mM MgCl2, and X-gal 1 mg/ml overnight at 37°C. Slides were post fixed with 4% paraformaldehyde before Sirius Red staining.

### Sirius Red Staining

Frozen and paraffin sections were stained with Picro-Sirius Red (Sigma, St. Louis, MO) for 1 hour and washed with acidified water until clear. Quantification of Sirius Red positive area was performed using Adobe Photoshop and NIH ImageJ software.

### Antibodies and Reagents

For immunohistochemistry and Western blots, primary antibodies used included: Id1 (Biocheck 1/37-2, Foster City, CA), Id3 (Ab 36501 Abcam, Cambridge, MA), αSMA (Ab 5694, Abcam) and clone 1A4 (Ab 7817, Abcam), Ki67 (Ab15580, Abcam), β-tubulin (clone TUB2.1, Sigma, St. Louis, MO), PDGFRβ (clone APB5, eBioscience, San Diego, CA), and VE-cadherin (550548, BD Bioscience). Sheep anti-rabbit and anti-mouse secondary antibodies used in Western blots were from GE Healthcare (Piscataway, NJ). Alexa Fluor goat anti-rat and anti- rabbit secondary antibodies were from Invitrogen.

### Western Blot

Western blots were performed as previously described [16] For quantification of protein levels, autoradiographs were scanned, and densitometry was performed with NIH ImageJ software. Results were corrected for variations in the amount of protein loaded on each lane using corresponding GAPDH or β -tubulin levels.

### Immunofluorescence and Immunohistochemistry

Immunofluorescence and immunohistochemistry were performed as previously described [16]. A mouse on mouse FITC kit (Vector Labs) was used for double labeling with Id1 (rabbit) and αSMA (mouse) antibodies. Quantification of capillary rarefaction was performed by overlaying a 10×10 grid using Adobe Photoshop and counting squares containing tubules with no CD31 signal. Quantification of PDGFRβ positive area was performed using Adobe Photoshop and NIH ImageJ software. For counts of double or single positive Id1 or Ki67 and PDGFRβ or VE-cadherin cells, 50–100 cells/HPF in 5 fields/kidney were identified. Id1 and Ki67 signal was correlated with overlying DAPI images to verify nuclear staining in individual cells. Cells with ambiguous correlation were not included in final cell counts.

To determine the tubular damage index (1–5), 10 microscopic fields (400x magnification) per H and E stained kidney section were assessed in a blinded fashion and tubular damage was graded as 1–5 with 1 = no damage, 2 = <25%, 3 = 25–50%, 4 = 50–75%, 5 = >75%. Results were expressed as means +/− SEM for 5 kidneys for each genotype at day 1 following IRI.

### Flow Cytometry

Isolation of endothelial cells was performed according to the method of Sobczak et al. [17] with the following modifications: livers were harvested from adult mice, the liver digest was purified by removal of macrophages with Dynabeads (Invitrogen, Grand Island, NY) coated with anti-CD16 antibody (BD Bioscience, San Jose, CA) for 20 minutes at room temperature, and positive selection was performed by incubation with anti-CD31 (BD Bioscience) coated Dynabeads for one hour at 4°C.

Endothelial cells were grown to 80% confluence in EGM-2 medium (Lonza, Walkersville, MD). They were then washed in PBS and disassociated with Accutase (Invitrogen). Cells were resuspended with in EGM with 5% FBS, and centrifuged at 150 g for 5 minutes. The pellet was then resuspended in FACS buffer (PBS, 5% FBS, 0.1% sodium azide, 2 mM EDTA) at a concentration of 500,000 cells per mL. 100 µL of cells were incubated with periodic agitation with 1 µL of fluorochrome-conjugated anti Flk-1 and PDGFRβ antibodies (BD Bioscience) for 45 minutes at 4°C. The cells were then washed twice with FACS buffer and resuspended in a final volume of 200 µL. Flow cytometry was performed using a MACSQuant Analyzer (Miltenyi Biotec, Bergisch Gladbach, Germany).

### Creatinine and Albumin Assays

Plasma creatinine was measured using an enzymatic kit (Abcam). Urine albumin was measured by ELISA (Exocell, Philadelphia, PA) and normalized to urine creatinine measured by Jaffe reaction (Creatinine Companion, Exocell).

### Statistical Analysis

Data were collected from independent experiments and presented as mean ± SEM. An unpaired two-tailed student’s *t*-test was used to compare 2 series of data. One way ANOVA for independent samples with a Tukey HSD test for comparison between 2 groups (www.vassarstats.net/anova1u.html) was used to compare 3 or more experimental groups. A p-value of less than 0.05 was considered significant.

## Results

**Figure 1:**
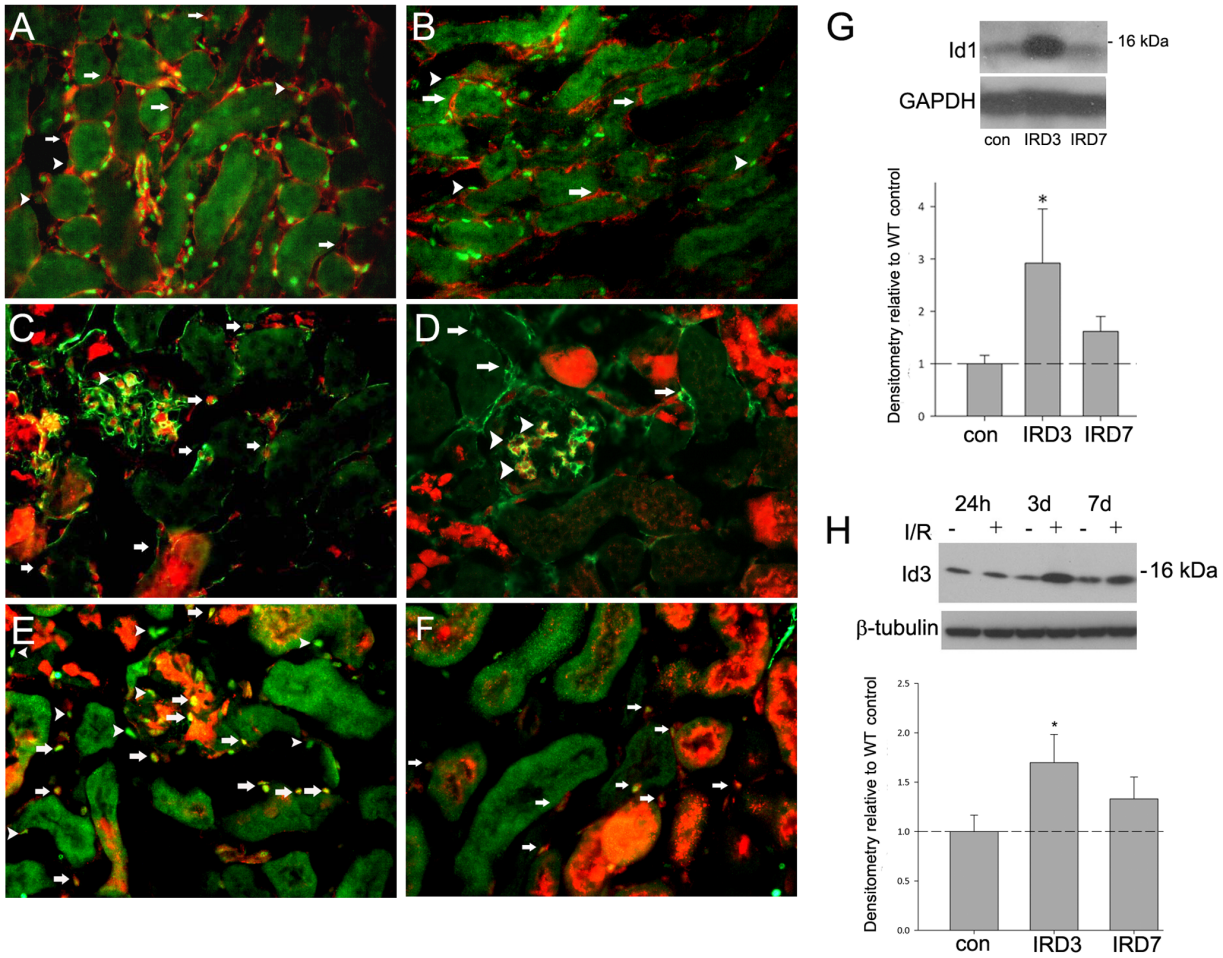
Id1 and 3 are co-localized in kidney endothelial cells and expression levels are transiently increased following ischemia-reperfusion injury (IRI). Immunofluorescence images for indicated antigens of normal (A–E) and 3 days post ischemia-reperfusion injury (F) kidneys from Id1+/+, Id3RFP/+ (C–F) and wild-type (WT) (A–B) mice. Red fluorescent protein (RFP) signal corresponds with Id3 expression. A) Green: Id1, red: CD31, arrows: Id1 negative, CD31 positive cells, arrowheads: Id1 low, CD 31 positive cells, B) Green: Id1, red: PDGFRβ, arrows: Id1 negative, PDGFRβ positive cells, C) Green: CD31, red: Id3RFP, arrows: double positive cells, D) Green: PDGFRβ, red: Id3RFP, arrows: single PDGFRβ positive cells, arrowheads: double positive mesangial cells, E) Green: Id1, red: Id3RFP, yellow: double positive cells in glomeruli (arrow) and interstitium (arrowhead), F) Green: Id1, red: Id3RFP, yellow: double positive cells in interstitium (arrows) 3 days following IRI, Original magnification: 400X. Representative Western blots of Id1 (G) and Id3 (H) expression following ischemia-reperfusion at indicated time points with corresponding densitometry from 5 mice for each time point. *p<.05 (unpaired two-tailed t-test).

Initial studies determined the baseline expression levels and localization of Id1 and Id3. Id3RFP knock-in mice (Id3RFP/+, GUDMAP consortium) express Red Fluorescent Protein from the Id3 locus and can therefore be used to detect endogenous Id3 expression. These mice and WT littermates were used to localize Id1 and Id3 by immunofluorescence in normal kidneys and following IRI using CD31 to label endothelial cells and PDGF receptor beta (PDGFRβ) to label pericytes and fibroblasts. In the normal adult kidney, Id1 was expressed at variable levels in CD31 positive peritubular endothelial cells in the cortex and medulla (Fig. 1A) and was absent from pericytes and other PDGFRβ positive interstitial cells (Fig. 1B). Id3 RFP signal was detected in endothelial cells (Fig. 1C) but was also present in glomerular mesangial cells (Fig. 1D) and in cortical and medullary collecting ducts. Id1 and Id3 co-localized in a subgroup of endothelial cells comprising 50–60% of cortical endothelial cells in normal kidneys (Fig. 1E). Examination of Id1 and 3 protein levels by Western blot showed that these proteins were increased 2–4 fold at day 3 following IRI and returned to baseline levels by day 7 (Fig. 1 G H). Id1 and 3 remained co-localized in peritubular endothelial cells following IRI (Fig. 1 F).

Following AKI from various causes including IRI, persistent capillary rarefaction contributes to chronic ischemia and fibrosis [18]. Based on the role of Id1 in promoting angiogenesis during development and in tumors, we sought to determine if overexpression of Id1 following IRI would prevent peritubular capillary loss and the development of fibrosis in comparison with WT littermates and Id1−/−, Id3RFP/+ mice. Id1−/−, Id3RFP/+ mice (referred to as Id1/Id3 KO) were produced by breeding Id1−/− mice (a gift from Dr. Robert Benezra) with Id3RPF/+ knock-in mice. Mice with doxycycline inducible endothelial Id1 expression were produced by breeding TRE Id1 mice (a gift from Dr. Robert Benezra) with Tg(Tek-rtTA, TRE-lacz) mice (Jackson Labs, rtTA under control of the Tie2 (Tek) promoter). Transgenic mice with only the Tek-rtTA, TRE-lacz allele have doxycyline inducible endothelial β-galactosidase expression [18], while double transgenic mice (referred to as TRE Id1) have both doxycycline inducible endothelial Id1 and β-galactosidase expression.

**Figure 2:**
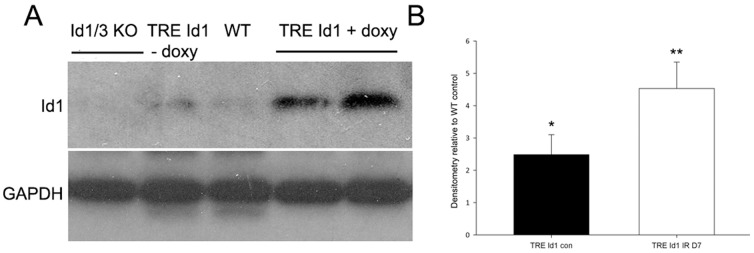
Transgenic mice have doxycycline inducible endothelial Id1 expression in normal kidneys and following IRI. A) Western blot of Id1 expression in control kidneys from mice with indicated genotype: Id1−/−, Id3RFP/+ = Id1/Id3 knockout (KO), wild-type littermate = WT, TRE Id1 = TRE Id1, Tie2 rtTA mice. Mice were fed doxycycline (+ doxy) for 1 week before isolation of kidney tissue or ischemia-perfusion injury. Control chow fed TRE Id1 mice (− doxy, lane 2) did not demonstrate ‘leaky’ Id1 expression compared with WT mice (lane 3). B) Densitometry of kidney Id1 levels from control (con) and day 7 following IRI (IRD7) TRE Id1 mice (n = 3/group) compared with WT control littermates. *p = .01, **p<.001 (unpaired two-tailed t-test).

By Western blot analysis, doxycycline fed double transgenic (TRE Id1) mice expressed 2–3 fold increased kidney Id1 levels compared with WT littermates or chow fed TRE Id1 mice (Fig. 2 A, B). Doxycycline induced Id1 expression was increased 3–5 fold at day 7 following IRI compared with WT littermates (Fig. 2 B).

**Figure 3:**
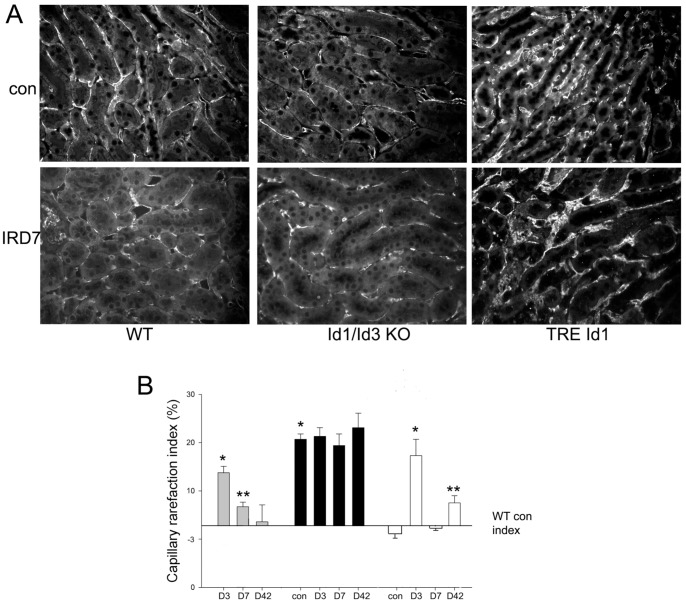
Id1/Id3 knockout mice have decreased baseline capillary density and Id1 transgenic mice have decreased capillary rarefaction 7 days following IRI followed by increased rarefaction at 42 days following IRI compared with WT mice. A) Immunofluorescence labeling of CD31 on outer medullary peritubular capillaries in normal kidneys (con) and at day 7 following IRI (IRD7) of indicated genotypes. B) Capillary rarefaction index (% area with no CD31 staining using 100 square grid) following IRI at indicated time points. *p<.001, **p = .04 compared with WT control kidneys (one way ANOVA with Tukey HSD test).

To determine the effect of Id1 expression levels on capillary density in normal kidneys and following IRI, baseline capillary density and areas of capillary rarefaction in the outer medulla, the location of the greatest tubular damage following IRI, were measured in WT, Id1/Id3 KO and TRE Id1 mice by CD31 labeling in normal mice and at 3, 7 and 42 days following IRI (n = 5/group, Fig. 3 A, B). As previously reported [19], WT mice had a 2-fold increase in areas of peritubular capillary rarefaction 3 days after IRI that persisted at significant levels at 7 days but recovered to baseline levels by 42 days following injury. Normal kidneys from Id1/Id3 KO mice displayed a nearly 3-fold increase in baseline capillary density compared with WT control mice that was not significantly changed at 3, 7 and 42 days following IRI. Normal kidneys from TRE Id1 mice had a non-significant decrease in baseline capillary density compared with WT control mice and developed peritubular capillary rarefaction 3 days after IRI. In contrast to WT mice, rarefaction resolved at 7 days following IRI but re-developed by 42 days following IRI.

**Figure 4:**
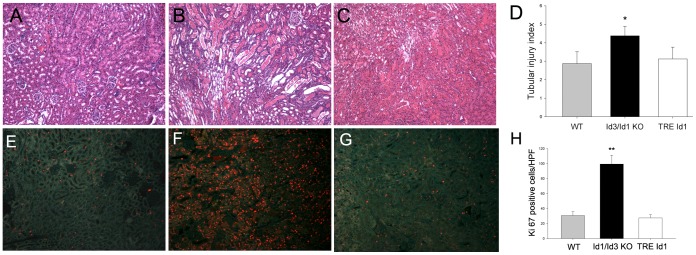
Id1/Id3 knockout mice have increased tubular damage following IRI compared with WT and Id1 transgenic mice. Hematoxylin and eosin staining of kidney sections from WT (A), Id1/Id3 KO (B) and TRE Id1 (C) mice at day 3 following IRI with corresponding assessment of tubular damage expressed as tubular injury index (1–5) (D) from 5 mice (mean +/− SEM) in each genotype *p<.01. Ki67 staining of from WT (E), Id1/Id3 KO (F) and TRE Id1 (G) mice at day 3 following IRI with corresponding cell counts (H) from 5 mice (Ki67 positive cells/HPF from 10 fields/kidney, mean +/− SEM) in each genotype *p<.001 (unpaired two-tailed t-test). Original magnification = 400X.

The effect of the observed changes in baseline capillary density on tubular injury following IRI was evaluated at day 1, the peak time of tubular injury [20]. As shown in Fig. 4, tubular injury was increased in Id1/Id3 KO mice compared with WT and TRE Id1 mice and extended to the proximal tubules throughout the outer cortex (A–D). The increased injury correlated with a 3–4 fold increase in proliferating proximal tubular cells labeled with Ki67 at 3 days following injury, the peak time of tubular repair (E–H). No significant differences in tubular injury or proliferation were noted in TRE Id1 mice.

**Figure 5:**
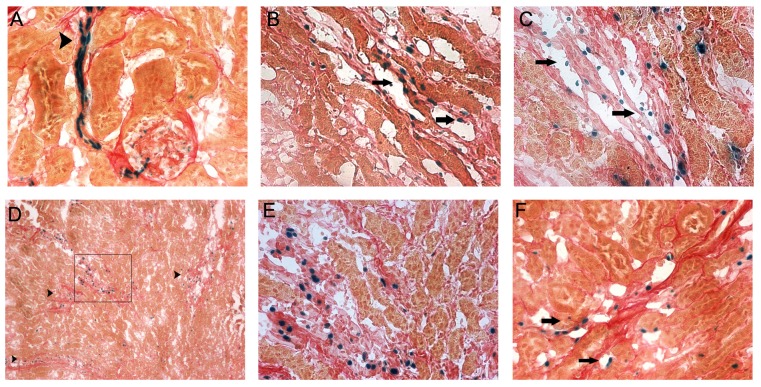
Doxycycline induced β-galactosidase expression is localized in interstitial cells in areas of collagen deposition in TRE Id1/Tie2-rtTA/TRE lacz mice but not in Tie2-rtTA/TRE lacz mice 7 days following IRI. Mice (n = 5/group) were fed doxycycline for indicated time prior to IRI. Mice were fed chow diets and sacrificed 7 days following IRI. X-gal (blue) followed by Picro Sirius Red staining for collagen of kidney cryosections for genotypes/doxycycline treatment: A) Control TRE Id1/Tie2-rtTA/TRE lacz mouse/doxycycline × 1 week, arrowhead: glomerular arteriole, B) IR day 7 TRE Id1/Tie2-rtTA/TRE lacz mouse, doxycycline × 1 week, arrows: capillary endothelial cells, C) IR day 7 TRE Id1/Tie2-rtTA/TRE lacz mouse, doxycycline × 2 weeks, arrows: capillary endothelial cells, D) IR day 7 TRE Id1/Tie2-rtTA/TRE lacz mouse, doxycycline × 4 weeks, arrowheads: area of medullary collagen deposition, E) High power magnification of area outlined in (D), F) IR day 7 Tie2-rtTA/TRE lacz mouse, doxycycline ×4 weeks, arrows: capillary endothelial cells. Original magnification: 400X (A–C, E–F), 100X (D).

The transgenic Tek-rtTA, TRE-lacz mice used in this study express β-galactosidase in cells expressing the doxycycline coupled transactivator (Tet-on system) under control of the Tie2 promoter. We examined X-gal staining as a marker to localize the areas of the kidney with doxycycline induced Id1 over-expression in double transgenic TRE Id1 mice under control conditions and following IRI. Kidneys were also stained with Sirius Red to identify collagen deposits following injury. In normal kidneys of doxycycline fed TRE Id1 mice, X-gal staining was most prominent in glomerular arterioles (Fig. 5A, arrowhead). Examination of kidneys at day 7 following IRI in TRE Id1 mice fed doxycycline for totals of 1, 2 and 4 weeks prior to surgery demonstrated that X-gal positive cells were predominately localized in peritubular capillary lumens in mice fed doxycycline for 1–2 weeks (B, C, arrows). Following more prolonged treatment, X-gal positive cells were localized in areas of medullary collagen deposition (D, low power image, arrowheads) and appeared to be located in areas of matrix outside of capillary lumens (E, high power image of area outlined in D). In contrast, X-gal positive cells remained within capillary lumens at 1 week following IRI in single transgenic Tek-rtTA, TRE-lacz mice fed doxycycline for 4 weeks, suggesting that Id1 over-expression in these Tie2 expressing cells induces cell migration away from capillary lumens. No X-gal positive cells were detected in control or post IRI kidneys in Tek-rtTA, TRE-lacz mice fed normal chow (data not shown).

**Figure 6:**
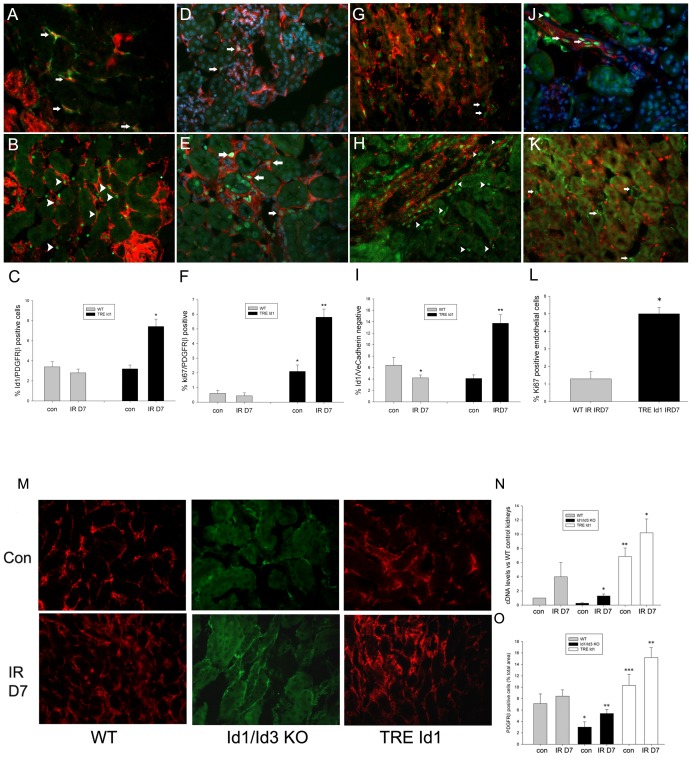
TRE Id1 mice have increased fibroblast Id1 expression and proliferation following ischemia-reperfusion injury. Immunofluorescence images for indicated antigens of normal (Con) and 7 days post ischemia-reperfusion (IRD7) kidneys from WT, Id1+/+, Id3 RFP/+ and TRE Id1 mice as indicated and corresponding cell counts from 5 mice/group. Cells from 5 sections from each group were counted and results displayed as means +/− SEM. A) Id1+/+, Id3RFP/+ IRD7: green: PDGFRβ, red: Id3RFP, arrows: double positive cells, B) TRE Id1 IRD7: green: PDGFRβ, red: Id1, arrowheads: double positive cells, C) Id1 positive/PDGFRβ positive cell counts (%) in normal and IRD7 WT vs. TRE Id1 mice, *p = .001, D) WT IRD7: green: Ki67, red: PDGFRβ, arrows: double positive cells E) TRE Id1 IRD7: green: Ki67, red: PDGFRβ, arrows: double positive cells F) Ki67 positive/PDGFRβ positive cell counts (%) in normal and IRD7 WT vs. TRE Id1 mice, *p = .003, **p<.001, G) WT green:Id1, red: VE-cadherin, arrows: single Id1 positive cells, H) TRE Id1 control: green: Id1, red: VE-cadherin, arrowheads: single Id1 positive cells I) Id1 positive/VE-cadherin negative cell counts (%) in normal and IR D7 WT vs. TRE Id1 mice, *p = NS, **p<.001, J) TRE Id1 IRD7: green: Ki67 (arrows: arteriole, arrowhead: peritubular capillary), red: VE-cadherin, K) TRE Id1 IRD7: green: αSMA (arrows), red: Id1, L) Ki67 positive endothelial cell counts (%) in normal and IRD7 WT vs. TRE Id1 mice, *p = .01 (unpaired two-tailed t-test). M) Immunofluorescence labeling for PDGFRβ in control and day 7 IRI kidneys sections from indicated genotypes, A–M) Original magnification: 400X. N) Comparative PCR for PDGFRβ levels in control and day 7 IRI kidneys sections from indicated genotypes. *p<.05 vs con, **p<.01 vs WT con. O) Quantification of PDGFRβ positive cells as a percentage of total tissue area in control and day 7 IRI kidneys sections from indicated genotypes *p<.05 vs WT con, **p<.05 vs con, ***p<.01 vs WT con (one way ANOVA with Tukey HSD test).

To determine the effects of Id1 over-expression on changes in interstitial cell proliferation and matrix formation following IRI, Kidneys were labeled with PDGFRβ to identify fibroblasts and pericytes or VE-cadherin to label endothelial cells and cell numbers in the outer medulla were compared between control day and 7 following IRI in TRE Id1 and WT littermate mice (n = 5 mice for each group). As shown in Figure 6, following IRI, Id3RFP (A, arrows) and Id1 positive nuclei (B, arrows), colocalize with peritubular PDGFRβ expressing cells. Quantification of double positive Id1/PDGFRβ cells demonstrated a 2–3 fold increase in TRE Id1 mice following IRI compared with no significant change in WT mice (C). Comparison of PDGFRβ proliferation by Ki67 labeling showed a corresponding 3-fold increase following IRI in TRE Id1 with no significant change in WT mice (D–F). The increase in Id1/PDGFRβ cells correlated with a 3–4 fold increase in Id1 positive/VE-cadherin negative cells (H, arrows) following IRI compared with no significant change in WT mice. Endothelial cell proliferation following IRI was also increased in TRE Id1 mice (J, L). No id1 positive nuclei were identified in αSMA expressing myofibroblasts following IRI (K), suggesting that Id1 may only be expressed in precursors for these cells.

PDGFRβ positive cell proliferation following IRI was further quantified by measuring the area of cell staining compared to total tubular area in the outer medulla (Fig. 6 M, O). PDGFRβ positive cell area in control kidneys correlated with baseline capillary density as measured in Fig. 3. PDGFRβ positive cell area was significantly increased in Id1/Id3 KO and TRE Id1 kidneys following IRI with no change observed in WT kidneys. The percent increase was similar in Id1/Id3 KO and TRE Id1 kidneys with the absolute area values highest in the TRE Id1 group. These results correlated with assays of kidney PDGFRβ cDNA levels by comparative qPCR (Fig. 6 N).

**Figure 7:**
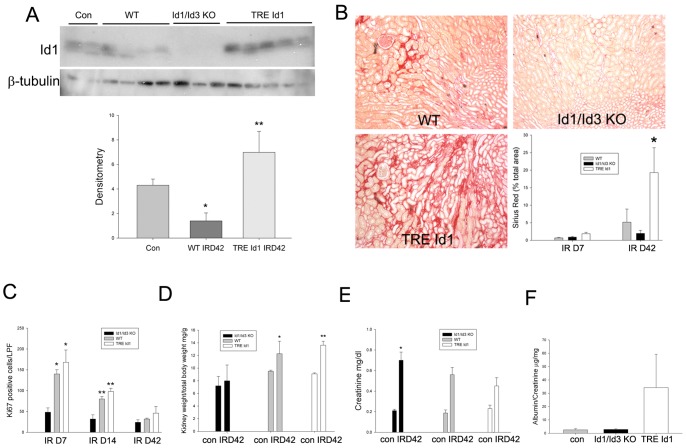
Medullary collagen deposition in TRE Id1/Tie2-rtTA mice is increased at 42 days following IRI compared with WT mice. A) Western blot of Id1 expression and corresponding densitometry in WT control (lanes 1–2), WT day 42 post IRI (lanes 3–6), Id1/Id3 KO day 42 post IRI (lanes 7–9) and TRE Id1 day 42 post IR (lanes 10–14) with β-tubulin as loading control *p<.05, *p<.001 B) Representative Sirius Red stained sections of WT, Id1/Id3 KO and TRE Id1/Tie2-rtTA mice at 42 days following IRI and comparison of areas of collagen deposits shown as mean +/− SEM for 5–7 mice/genotype group. *p = .05 compared with TRE Id1 control. C) Ki67 positive peritubular interstitial cells (nuclei/low power field: 40X) at indicated time points following IRI. *p = .003, **p = .01 compared with WT control. D) Kidney weight (mg) normalized to total body weight (grams) at 42 days following IRI compared with contralateral kidney weights at nephrectomy/ischemia surgery. *p<.01, **p<.0001. E) Plasma creatinine (means +/− SEM) levels for control mice and 42 days following IRI *p = .05 for Id1/Id3 KO vs. TRE Id1 mice (unpaired two-tailed t-test). F) Urine albumin/creatinine ratios for spot urine samples at 42 days following IRI.

Mice were examined at 42 days following IRI and contralateral nephrectomy to determine the effect of changes in Id expression on kidney regeneration and the long-term progression of fibrosis. At 42 days following IRI, Id1 levels were decreased by 50% in WT mice compared with sham operated mice. Id1 levels in TRE Id1 mice fed doxycycline during this time period were increased 7-fold compared with WT littermates (Fig. 7A). Decreased Id1 levels correlated with a decrease in Id1 expressing endothelial cells from 89+/−2.4% to 29+/−8.3% (p = .002) by immunofluorescence co-localization of Id1 and CD31 (data not shown). The development of kidney fibrosis following IRI was compared using Sirius Red staining for collagen deposition (Fig. 7B). Areas of collagen deposition in the inner cortex and outer medulla were increased 2–3 fold at 7 days following IRI in TRE Id1 compared with WT and Id1/3 KO kidneys and were subsequently increased 5 fold in WT and 18 fold in TRE Id1 mice. In contrast, Id1/Id3 KO mice had no significant increase in collagen deposition at these 2 time points. Examination of cell proliferation using Ki67 immunofluorescence demonstrated significantly increased numbers of proliferating peritubular interstitial cells in WT and TRE Id1 mice at 7 and 14 days following IRI compared with Id1/Id3 KO mice, and no significant difference at 42 days (Fig. 7C).

Following unilateral nephrectomy, the contralateral kidney compensates for the reduced nephron number by hypertrophy of tubular epithelial cells and glomeruli, resulting in preservation of GFR [21]. Following IRI and contralateral nephrectomy, WT and TRE Id1 mice developed a 30–40% increase in kidney weight at 42 days (Fig. 7D), consistent with previous reports [22]. In contrast, Id1/Id3 KO mice had no increase in kidney weight, resulting in a significant increase in creatinine compared with TRE Id1 mice (Fig. 7E). Id1/Id3 KO mice had no albuminuria despite an increase in creatinine, consistent with the absence of fibrosis. In contrast, TRE Id1 mice had elevated albuminuria, correlating with increased fibrosis (Fig. 7F).

**Figure 8:**
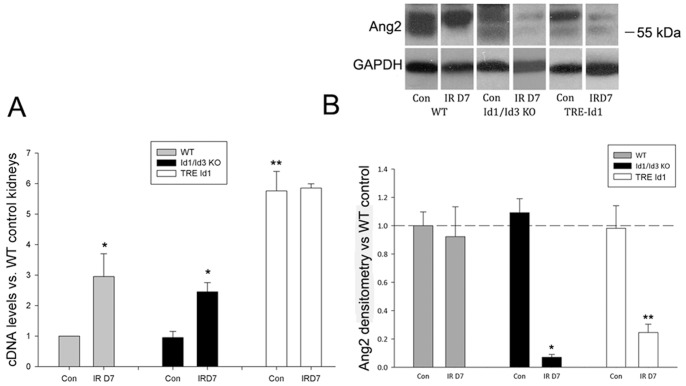
Angiopoietin 1 levels are increased in TRE Id1 mice and Angiopoietin 2 levels are decreased following IRI in knockout and transgenic mice. A) Comparative PCR expressed as levels relative to WT control kidneys for Ang1 levels in control and day 7 post IRI kidneys of WT, Id1/Id3 KO, and TRE Id1 mice. *p<.05, **p<.01. B) Western blot of Ang2 levels in control and day 7 post IRI kidneys of WT, Id1/Id3 KO, and TRE Id1 mice. Results expressed as levels relative to WT control kidneys. *p<.001, **p<.05. (one way ANOVA with Tukey HSD test).

The changes in vascular remodeling including perivascular cell proliferation following kidney injury in KO and transgenic kidneys, suggested the possibility that the observed differences could be due to effects on Angiopoietin (Ang) levels. The Tie2 receptor agonist Ang1 and antagonist Ang 2 are the primary regulators of vascular remodeling in both embryos and adult tissues following injury [23]. To determine the effect of changes in Id levels in control kidneys and following IRI, Ang1 expression was assayed by comparative qPCR (Fig. 8A) and Ang 2 was assayed by Western blot (Fig. 8B). Ang 1 levels were significantly increased in WT and KO kidneys following IRI as previously reported [24]. Baseline Ang 1 levels in TRE Id1 mice, however, were increased 5–6 fold compared to WT control kidneys, with no significant increase following IRI. Ang 2 levels were similar at baseline in all three genotypes but were significantly decreased in both KO and TRE Id1 kidneys following IRI, in contrast to unchanged levels in WT mice.

**Figure 9:**
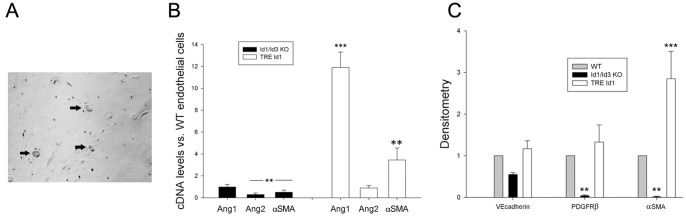
MIcrovascular cell cultures from TRE Id1 mice express increased Ang1 and αSMA. Cells from livers of WT, Id1/Id3 KO, and TRE Id1/Tie2-rtTA mice (n = 5 for each genotype) were isolated using CD31 microbeads and cultured until 70–80% confluent in endothelial basal medium. TRE Id1/Tie2-rtTA cells were treated with 1 µg/ml doxycyline for 3 days prior to assays. A) X-gal staining of TRE Id1/Tie2-rtTA cells demonstrating positive nuclei (arrows). B) Comparative PCR for Ang1, Ang 2 and αSMA for Id1/Id3 KO, and TRE Id1/Tie2-rtTA relative to WT cells. **p<.05, ***p<.01. C) Western blot of VE-cadherin, PDGFRβ and αSMA levels in Id1/Id3 KO and TRE Id1/Tie2-rtTA cells. Results expressed as densitometry levels relative to WT cells. **p<.001. ***p<.01. (one way ANOVA with Tukey HSD test).

Because of the complex cellular environment of the ischemic kidney including injured tubules, infiltrating inflammatory cells and proliferating fibroblasts, we next determined changes in gene expression associated with Id levels in microvascular cells isolated from genotypes used in the above *in vivo* experiments. Microvascular cells were prepared from mouse livers (the only organ in the adult mouse that we found to yield adequate numbers of viable cells for further analysis) of WT, KO and TRE id1 mice (n = 4/group) using CD31 microbeads and cultured in endothelial basal medium. By flow cytometry, cell preparations (n = 10 mice) contained an approximately equal number of Flk-1 positive endothelial cells and co-precipitated PDGFRβ positive cells that may represent fibroblasts or hepatic stellate cells/pericytes. Cells from TRE Id1 mice were treated with doxycycline (1 µg/ml) for 3 days prior to isolation of RNA and protein. X-gal staining of treated cells showed that a small percentage (2–5%) of these cells expressed the Tek-rtTA, TRE-lacz transgene (Fig. 9 A, arrows). Examination of Ang1 and 2 expression by comparative qPCR (Fig. 9B) demonstrated that Ang 1 levels were increased 12-fold in TRE Id1 cells compared with WT cells and were unchanged in KO cells, correlating with PCR results from whole kidneys. Western analysis of the endothelial marker VE-cadherin and mesenchymal markers PDGFRβ and αSMA (Fig. 9C) showed no differences in VE-cadherin levels but significant reductions in mesenchymal markers in KO cells and a significant increase in αSMA in TRE Id1 cell cultures, correlating with PCR results.

## Discussion

Id proteins have diverse functions in development and tumor growth and have been shown to regulate proliferation and differentiation of epithelial, endothelial and vascular smooth muscle cells during these processes. Expression levels are generally decreased in adult organs and are increased with inflammation and tumor formation where Id1 and Id3 have been shown to have a key role in promoting angiogenesis. The effect of Id1 and 3 expression levels on angiogenesis and microvascular remodeling following adult tissue injury has not been a subject of investigation with the exception of studies of bleomycin induced lung injury [25] that showed increased endothelial cell apoptosis and lung fibrosis in Id1 knockout mice and dermal wound healing [26] that showed impaired angiogenesis and granulation tissue formation in endothelial specific Id1 and Id3 knockout mice. Our study showed no fibrosis in Id1/Id3 KO mice and decreased Id1 levels in WT mice with minimal fibrosis at 42 days following IRI and increased fibrosis following IRI in mice with inducible endothelial Id1 expression, suggesting that decreased Id1 levels following injury may protect against the development of fibrosis. Reasons for the differences in this study and the previous lung study are not clear but may be due to the mechanism of injury or organ specific factors.

Endothelial Id1 expression is higher in kidney tissue compared to other tissues we examined including lung, liver, skin and white fat. The relatively high Id levels may be due to increased BMP7 production in the kidney compared to other tissues [27]. Increased Id1 expression may maintain the integrity of the microvasculature in the relatively hypoxic kidney environment. This study demonstrates that Id1−/−, Id3+/− mice have decreased kidney capillary density resulting in greater tubular damage following IRI. Despite this deficit, KO kidneys displayed normal reparative tubular cell proliferation with no evidence of increased fibrosis or capillary rarefaction over baseline levels, though baseline levels were reduced to levels found in WT mice following IRI. Interestingly, KO mice had no compensatory kidney hypertrophy and hyperplasia following contralateral nephrectomy resulting in a 3 fold increase in creatinine at 42 days following IRI. No Id1 or 3 expression was detected in proximal tubule cells in WT mice, the primary cell type that undergoes hypertrophy following nephrectomy [21], suggesting that altered paracrine signaling by surrounding endothelial or interstitial cells may be responsible for this difference. Inducible overexpression of Id1 in endothelial cells resulted in reduced capillary rarefaction during the initial repair phase. This was, however, associated with increased interstitial fibroblast proliferation, matrix deposition and proteinuria at a later time point. Future studies are necessary to determine if secretion of Id dependent endothelial paracrine factors result in excess matrix production and cell hypertrophy or hyperplasia that may result in tissue fibrosis. The results from this study also suggest that decreasing Id protein expression several days following AKI and in chronic renal failure may be a therapeutic target to prevent the development of kidney fibrosis.

In this study, overexpression of Id1 depends on transgenic expression of the tetracycline transactivator rtTA under control of the Tie2 promoter. Previous studies have shown that Tie2 is most highly expressed in glomerular tufts, cortical peritubular capillaries and the medullary vasa rectae in postnatal mouse kidneys [28]. X-gal staining of kidneys for Tek-rtTA, TRE-lacz transgene expression showed the greatest number of positive cells in glomerular arterioles, peritubular capillaries in the medulla and vasa rectae. The Tie2 (Tek) rtTA mice used in this study were predicted to have the highest levels of doxycycline induced Id1 expression in these areas. While Id1 immunofluorescence is not a quantitative method to detect changes in expression in doxycycline treated mice, the highest number of proliferating endothelial cells and Id1/PDGFRβ double positive cells were found near medullary peritubular capillaries and in the vasa rectae. We found much lower Tie2/β-galactosidase expression in other organs including the lung and liver, a difference that may be due to the relative hypoxic environment of the kidney [29].

Following IRI, X-gal positive cells in TRE Id1 double transgenic but not single Tek-rtTA, TRE-lacz mice were observed to migrate away from capillary lumens in areas of collagen deposition that was dependent on increased time of doxycycline treatment from 1 to 3 weeks. Increased numbers of Id1/PDGFRβ double positive cells were also observed in areas of fibrosis following IRI in TRE Id1 mice along with increased numbers of VE-cadherin negative Id1 positive cells. Together, these results suggest the possibility that over-expression of endothelial Id1 may lead to mesenchymal transition to a fibroblast progenitor cell and resulting fibrosis following injury [30], [31]. Id1 was not detected in any αSMA positive cells, suggesting that Id1 downregulation may be necessary for myofibroblast differentiation from induced precursor cells. We were unable to clearly label cells for mesenchymal markers following X-gal staining and antibodies against β-galactosidase have been shown to be unreliable in kidney sections [2]. More definitive proof of this possibility will require development of mice with both inducible Id1 over-expression and a specific endothelial lineage marker.

An alternative explanation for the observed effects of Id1 over-expression in endothelial cells may be changes in paracrine signaling between endothelial cells and adjacent mesenchymal cells. This possibility is supported by microvascular cell cultures containing a mixture of endothelial and PDGFRβ positive cells that demonstrated a marked increase in αSMA levels despite small numbers of β-galactosidase expressing cells and very low levels in KO cells.

Angiopoietins are ligands of the Tie2 receptor tyrosine kinase and regulate vascular remodeling during embryogenesis and following tissue injury [23]. In adult mice, perivascular cell secreted Ang1 promotes endothelial quiescence and vascular stability. Ang1 attenuates capillary loss and induces formation of enlarged capillaries during development and following injury. Endothelial Ang2 expression is increased in activated and hypoxic tumor endothelial cells where it antagonizes Ang1 and causes vascular instability. Since kidney VEGF levels are reduced following IRI [30], Ang2 would be expected to have an anti-angiogenic effect. In the postnatal mouse kidney, knockout of Ang 2 results in increased numbers of αSMA expressing pericytes [24], a result that may be due to unopposed Ang1 effects. These findings suggest the possibility that the increased numbers of PDGFRβ expressing pericytes in TRE Id1 mice following IRI may be a consequence of increased Ang1 and decreased Ang2 levels resulting in unopposed endothelial Tie2 signaling. These results are consistent with a previous study of mice with AKI secondary to folic acid administration that were shown to have decreased capillary rarefaction following treatment with recombinant Ad-Ang1 but developed increased fibrosis, an effect that may have been due to abnormal endothelial-mesenchymal cell interaction with increased resident fibroblast proliferation [32]. The results from this study are also consistent with previous studies demonstrating that endothelial overexpression of Id1 results in increased [33] and Id1 and 3 knockout results in decreased Ang 1 levels [26]. Alternative explanations for the increased Ang1 levels in TRE Id1 mice may be that the increased numbers of pericytes and fibroblasts in these mice result in greater production of Ang1 or that increased Ang1 levels are compensatory for increased angiogenesis following injury [34]. Additional studies using co-cultures of endothelial cells and pericytes from these mice will be necessary to determine the actual mechanism.

Our results also show that both Id1 knockout and transgenic Id1 expression result in decreased Ang 2 levels following IRI. This contradictory result may due to difference in the mechanism of regulation of Ang2 in the 2 different genotypes. Id1 KO mice have been shown to have reduced inflammatory responses [14], [35] that may reduce the release of Ang2 from endothelial or inflammatory cells while TRE Id1 mice may have reduced levels due to reduced tissue hypoxia.

Results from this study may be applied to other processes associated with increased endothelial Id1 expression including tumor angiogenesis. Id1 expression has been shown to promote tumor angiogenesis by increasing endothelial progenitor cell formation [36]. It may be of interest to determine if the increased Ang1 expression and fibroblast proliferation noted in this study also occurs in tumor vessels with increased Id1 levels resulting in a more mature and stable vasculature. In chronic kidney disease and fibrosis, BMP7 levels are decreased, suggesting a contribution to fibrosis that we speculate may be due, in part, to decreased endothelial Id levels and capillary rarefaction.
